# First person – Shohei Yoshimoto

**DOI:** 10.1242/dmm.047068

**Published:** 2020-09-28

## Abstract

First Person is a series of interviews with the first authors of a selection of papers published in Disease Models & Mechanisms, helping early-career researchers promote themselves alongside their papers. Shohei Yoshimoto is first author on ‘Inhibition of Alk signaling promotes the induction of human salivary-gland-derived organoids’, published in DMM. Shohei is a research scientist in the lab of Professor Shuichi Hashimoto at Fukuoka Dental College, Fukuoka, Japan, investigating salivary gland disorders.


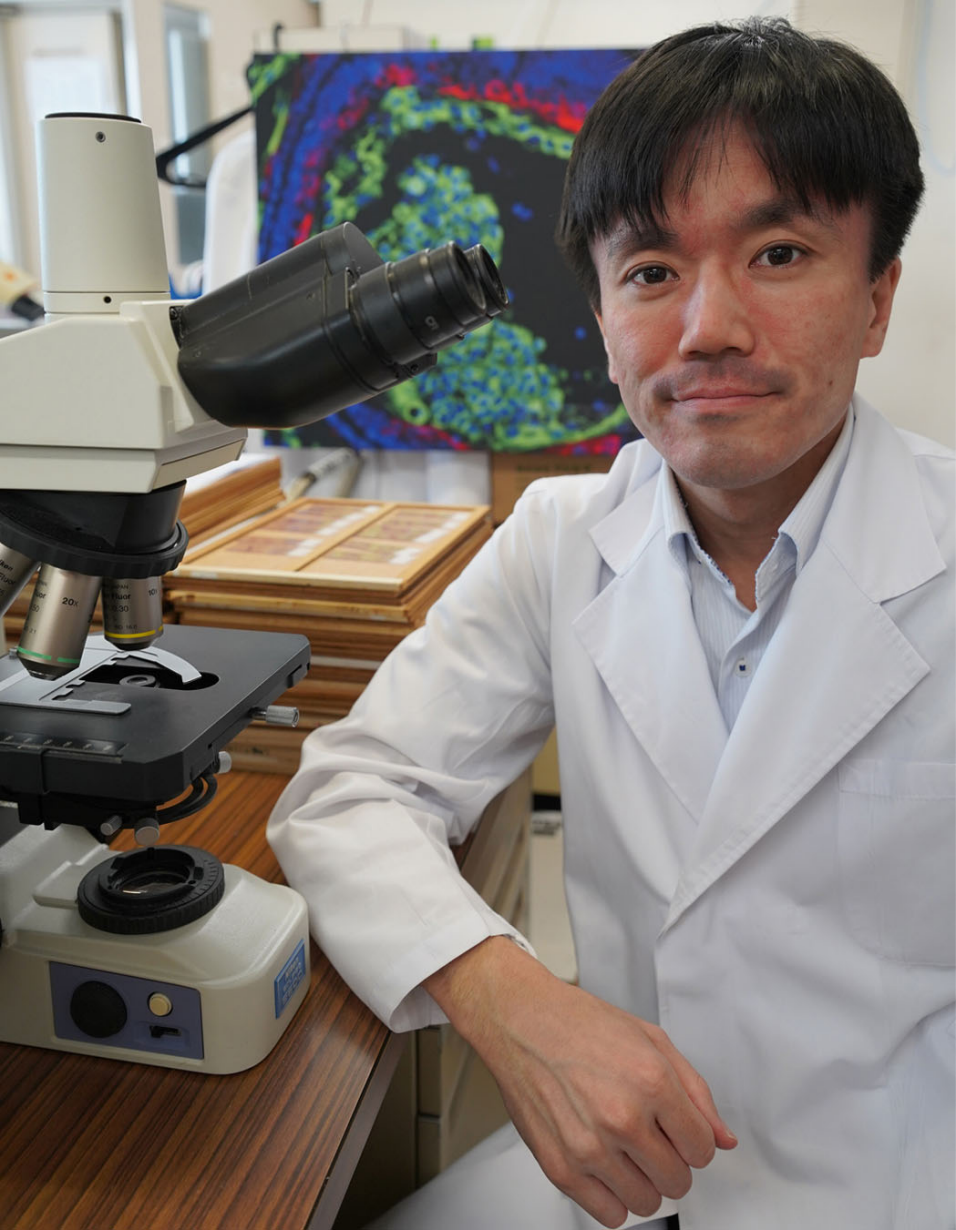


**Shohei Yoshimoto**

**How would you explain the main findings of your paper to non-scientific family and friends?**

Hyposalivation is a cause of several morbidities, including dental caries, painful mucositis, oral fungal infections, sialadenitis and dysphagia. However, the mechanisms underlying hyposalivation and the potential therapeutic targets are not fully understood. To clarify the mechanisms of salivary gland dysfunctions, some useful tools, such as culture systems to produce ‘organoids’ that mimic the human salivary glands, are desired for analysis of the morphological and functional changes involved in salivary gland dysfunction. In this study, we established a culture system to produce human salivary-gland-derived organoids, then we revealed the usefulness of this culture system by applying it in assays to study hyposalivation.

**What are the potential implications of these results for your field of research?**

The organoid system reported in our article could be of benefit to *in vitro* analyses of the morphological and functional changes associated with salivary gland dysfunctions in several research fields, such as pathobiology, inflammation and regenerative medicine.

**What are the main advantages and drawbacks of the model system you have used as it relates to the disease you are investigating?**

The main advantage of the model system is that our organoid model was established from human salivary gland tissue. Human-tissue-derived organoids are potential models for drug screening systems and regenerative medicine. A drawback of the organoid system is that we needed human salivary gland tissue to make the organoids.

“When we successfully induced organoid swelling […], we were surprised and excited.”

**What has surprised you the most while conducting your research?**

To evaluate if our organoid system could be applied for the analysis of some actual salivary gland diseases or disorders as an experimental system, we established a swelling model. When we successfully induced organoid swelling, revealing the mechanism of saliva secretion affected by the stimulation, we were surprised and excited.

**Figure:**
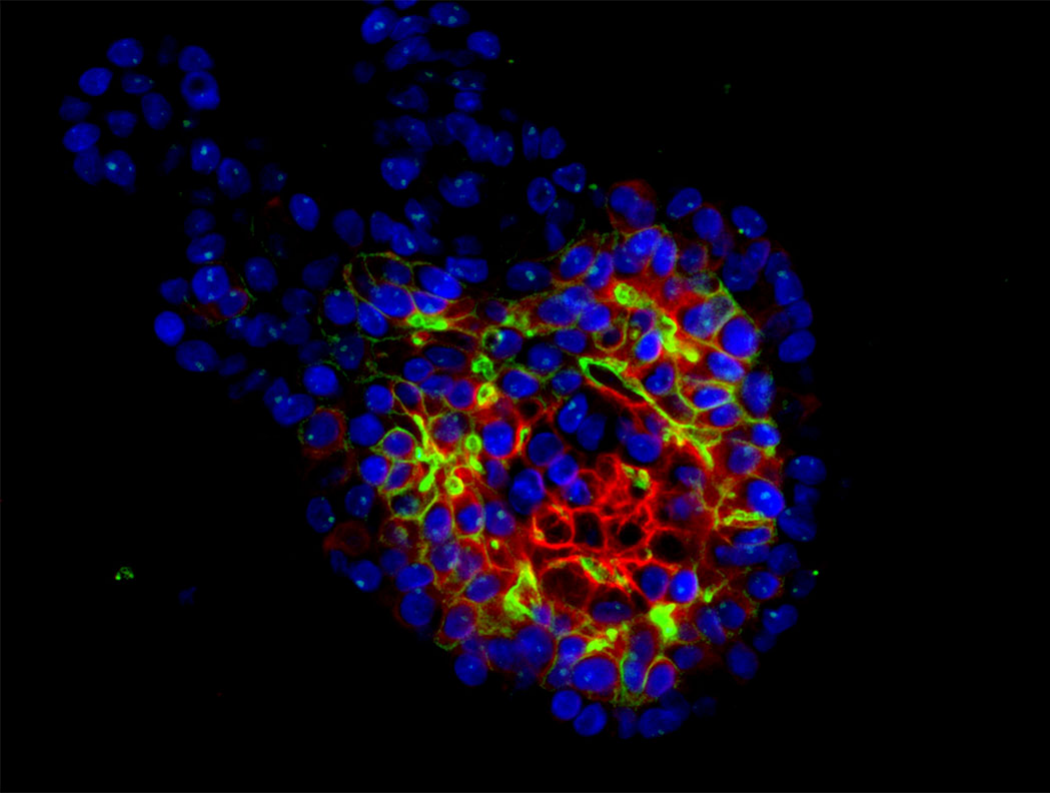
**Immunofluorescence staining of human salivary-gland-derived organoids.** Staining for aquaporin 5 (green) and keratin 18 (red), markers of acinar cells, is seen in the inner cells of organoids. Nuclei are stained with DAPI (blue).

**Describe what you think is the most significant challenge impacting your research at this time and how will this be addressed over the next 10 years?**

Organoid culture systems have been established from cells of several organs, and have become useful as disease models in several research fields, including pathobiology, developmental biology, inflammation, regenerative and cancer medicine, and drug discovery. We need to meet the challenge of establishing automatic culture systems for approaching translational research goals.

“Due to the COVID-19 pandemic, it is difficult for early-career scientists to study abroad. On the other hand, we can now easily attend many web conferences […] and can make new networks of researchers.”

**What changes do you think could improve the professional lives of early-career scientists?**

Due to the COVID-19 pandemic, it is difficult for early-career scientists to study abroad. On the other hand, we can now easily attend many web conferences, seminars and workshops, and can make new networks of researchers. We need to adapt to these changes and accelerate our own research.

**What's next for you?**

My next challenge is to investigate unknown mechanisms in salivary gland disorders. I want to discover selective drugs for salivary gland diseases.
